# School Hygiene

**Published:** 1905-01-14

**Authors:** 


### The Hospital.

A Journal of

The Medical Sciences and Hospital Administration.

Vol. XXXVII.—No. 955. JANUARY 14, 1905.

### School Hygiene.

The Conference on School Hygiene, to which we directed attention in our last issue, and which will be held in the great hall of the University of London on February 7th and the three following days, will, it may be hoped, not only be numerously attended, but attended by a class of people able, by their position and influence, to bring the conclusions of the conference into practical operation in the schools. Much attention has lately been bestowed upon the prevailing idea that there is a marked tendency to physical degeneration among the lower strata of the English people; and, whether this idea be correct or not, it is at least certain, as Sir Lauder Brunton set forth in one of the two addresses which he delivered last week, that there is a large amount of failure to reach what might be described as a satisfactory standard of bodily development. It is necessary, indeed, to confess that the expectations which were excited in some minds by the passage of the first Elementary Education Act have not been realised; and that the last 30 years have witnessed deterioration rather than improvement, alike in the physical, the moral, and the intellectual standards of our juvenile population of the lower working classes. The sweeping together of the children into schools has greatly promoted the occurrence of epidemic outbreaks of disease, by which, in a large number of instances, enduring bodily weakness has been entailed upon the sufferers. The teaching, in so far as it has aimed at conferring attainments upon the pupils, has in many instances been greatly in excess of their powers of useful assimilation; and in others has been in excess of their opportunities of applying what they were taught; while nearly all that is included within the meaning of the good old West-country word "behaviour" has been suffered to perish from neglect. We have established an enormous and very costly machinery in every part of the kingdom, and, if we look for its results, we find no appreciable improvement in either the moral or the intellectual position of the classes whom it was intended to benefit. The power of reading is chiefly exercised upon matter which it would be better to leave unread, the more dishonest aspects of trades-unionism were never more strongly in evidence, the earnings of the labouring classes were never diverted in larger proportion by the publican and the tobacconist, the shambling and narrow-chested loafer was never more conspicuous, and paupers and the professionally "unemployed" are annually increasing in number. Sir Lauder Brunton has declared, we think quite correctly, that it is hopeless to attempt any improvement of adults, and that our only prospect is with the children. If this be so, it is at least certain that we must devise some better methods of approaching and of dealing with them.

Rather more than 200 years have passed away since Locke wrote that "Children's time should be spent in acquiring what may be useful to them when they come to be men"; but this aphorism, or, as it might almost be described, this truism, has never asserted its proper sway over the minds of so-called educators. They have endeavoured to use learning as a gynæcologist uses a sponge tent, and have sought, by the introduction of compressed facts, mechanically to expand the intellect. There has been a fallacious idea of "elevating" the masses by giving them instruction of a kind, useful and necessary for those higher in the social scale, but altogether useless in the lower strata of life. If we are to carry out Sir Lauder Brunton's view, and to improve our poorer children, we shall not do so by teaching the boys mathematics and the girls to play on the pianoforte, but by trying to render both sexes more clean, more civil, more healthy, more intelligent, and, in the homely language of the Catechism, more fit for that state of life unto which it has pleased God to call them. The social evils of this country are largely due to the circumstance that political power has passed to the working classes before they have been sufficiently educated to exercise it wisely, with the natural consequence that the professional politicians who appeal to them find it the least trouble to base their appeals upon false issues. We may reasonably hope that the disorders hence arising will right themselves in time; but they will not be corrected by any endeavour to force a progress which, in the essential nature of things, must be gradual if it is to be effective. The *corpus sanum* must precede the *mens sana*, and, if we really wish to elevate our labouring classes through their children, we must first direct our teaching towards the attainment and preservation of bodily health and vigour, to which all other things may be added hereafter. We must not allow diphtheria to become epidemic, and a hundred children to be permanently injured by it, while a pedantic "education authority" is resisting the efforts of the medical officer of health to close a school. We should spare no pains to teach the elder girls something of the domestic conditions conducive to health, something of the value of ventilation, something of the properties of different kinds of food. In this last respect we unquestionably owe a debt to the poor. We have taught them to read, but we have not taught them the rudiments of judgment, and we have left them a helpless prey to the wiles of the common advertiser. Our walls are covered with posters setting forth the alleged merits of this, that, and the other food-preparation, and such preparations are largely bought by the poor. We think it should be the business of municipalities to enlighten the ignorant concerning the actual worth of the preparations so sedulously forced upon their notice. Something of this kind is done in many foreign countries, and we should have fewer starved and rickety children if it were done also in England.

